# Lactadherin immunoblockade in small extracellular vesicles inhibits sEV-mediated increase of pro-metastatic capacities

**DOI:** 10.1186/s40659-023-00477-8

**Published:** 2024-01-03

**Authors:** Eduardo Durán-Jara, Matías del Campo, Valentina Gutiérrez, Ignacio Wichmann, César Trigo, Marcelo Ezquer, Lorena Lobos-González

**Affiliations:** 1 Center for Regenerative Medicine, Institute for Sciences and Innovation in Medicine, Facultad de Medicina, Clínica Alemana Universidad del Desarrollo, Santiago, Chile; 2 Facultad de Medicina, Clínica Alemana Universidad del Desarrollo, Santiago, Chile; 3 Division of Obstetrics and Gynecology, Department of Obstetrics, Escuela de Medicina, Pontificia Universidad Católica de Chile, 8331150 Santiago, Chile; 4 Advanced Center for Chronic Diseases (ACCDiS), Independencia, Santiago, Chile

**Keywords:** Breast cancer, Extracellular vesicles, Lactadherin, Metastasis

## Abstract

**Background:**

Tumor-derived small extracellular vesicles (sEVs) can promote tumorigenic and metastatic capacities in less aggressive recipient cells mainly through the biomolecules in their cargo. However, despite recent advances, the specific molecules orchestrating these changes are not completely defined. Lactadherin is a secreted glycoprotein typically found in the milk fat globule membrane. Its overexpression has been associated with increased tumorigenesis and metastasis in breast cancer (BC) and other tumors. However, neither its presence in sEVs secreted by BC cells, nor its role in sEV-mediated intercellular communication have been described. The present study focused on the role of lactadherin-containing sEVs from metastatic MDA-MB-231 triple-negative BC (TNBC) cells (sEV-MDA231) in the promotion of pro-metastatic capacities in non-tumorigenic and non-metastatic recipient cells in vitro, as well as their pro-metastatic role in a murine model of peritoneal carcinomatosis.

**Results:**

We show that lactadherin is present in sEVs secreted by BC cells and it is higher in sEV-MDA231 compared with the other BC cell-secreted sEVs measured through ELISA. Incubation of non-metastatic recipient cells with sEV-MDA231 increases their migration and, to some extent, their tumoroid formation capacity but not their anchorage-independent growth. Remarkably, lactadherin blockade in sEV-MDA231 results in a significant decrease of those sEV-mediated changes in vitro. Similarly, intraperitoneally treatment of mice with MDA-MB-231 BC cells and sEV-MDA231 greatly increase the formation of malignant ascites and tumor micronodules, effects that were significantly inhibited when lactadherin was previously blocked in those sEV-MDA231.

**Conclusions:**

As to our knowledge, our study provides the first evidence on the role of lactadherin in metastatic BC cell-secreted sEVs as promoter of: (i) metastatic capacities in less aggressive recipient cells, and ii) the formation of malignant ascites and metastatic tumor nodules. These results increase our understanding on the role of lactadherin in sEVs as promoter of metastatic capacities which can be used as a therapeutic option for BC and other malignancies.

**Supplementary Information:**

The online version contains supplementary material available at 10.1186/s40659-023-00477-8.

## Introduction

Breast cancer (BC) is one of the most common cancers worldwide, leading both incidence and mortality rates [1]⁠. According to GLOBOCAN, it was estimated that more than 19 million new cases and more than 10 million deaths occurred during 2020 due to BC [1]⁠. Currently, even with scientific and technological advancements, both diagnosis and treatment are not effective, especially in the advanced stages of the disease. Moreover, the Covid19 pandemic is expected to have a negative impact on the diagnosis and treatment of patients [2–5]⁠. Thus, new and better biomarkers for BC diagnosis, prognosis, progression, and therapeutic targets are urgently needed to improve poor outcomes.

Considering breast tumor diversity and complexity [6]⁠, intratumor cellular communication and tumor/stromal cell intercommunication play a major role in modulating the invasive potential of malignant cells in the early and advanced stages of the disease. This complex cellular interplay can be carried out through direct contact or mediated by extracellular signals (autocrine, paracrine, juxtracrine or endocrine) [7]⁠. Recent studies have shown that, especially in metastatic stages, the main route of extracellular communication is mediated by small extracellular vesicles (sEVs; mainly exosomes and microvesicles), which have been proposed to play essential roles in the promotion of tumorigenic capacities and the preparation of metastatic niches [8–11]⁠. sEVs are membrane-derived EVs that are released by various cell types. These 40–200 nm diameter EVs play important roles in intercellular communication through the transfer of several bioactive molecules, such as nucleic acids (DNA, mRNAs, microRNAs, and other ncRNAs), proteins, and lipids, which can modulate the phenotype and function of a recipient cell [12]⁠, thus promoting tumor development, progression, and metastasis [13–16]⁠.

⁠sEVs have been proposed as promising diagnostic biomarkers, therapeutic targets and drug delivery vehicles for breast [17, 18]⁠ and other types of cancer [19–24].⁠ On the other hand, it has been shown that sEVs secreted by metastatic cells potentiate tumorigenic capacities of less aggressive neighbor recipient cells [8, 9, 13, 15, 16, 25–36]⁠. Different studies have shown that sEVs secreted by metastatic BC cells potentiate tumorigenic and metastatic potential when incorporated into recipient cells [9, 16, 25, 26, 33, 36]⁠. For instance, recent studies have shown that sEVs secreted by metastatic MDA-MB-231 TNBC cells promote the invasive potential and anchorage-independent growth capacity of cells with lower tumorigenic and metastatic potential such as MCF7 and T47D cells [26, 37]⁠. However, the specific molecules responsible for these phenotypic and functional changes have not been completely elucidated and can vary between different sEV populations and different cancer types.

Milk fat globule EGF and factor V/VIII domain containing protein (a.k.a. lactadherin), that in humans is encoded by the *MFGE8* gene, is a secreted glycoprotein associated with the milk fat globule membrane [38]⁠. Lactadherin was first described as a marker of BC progression in 1991 [39]⁠. A pro-tumorigenic function of lactadherin has been documented in several types of human cancer [reviewed in 40]⁠. Notably, it seems that lactadherin contributes to tumor progression [41–45]⁠, promotes survival of tumor cells [46, 47]⁠, induces epithelial-mesenchymal transition (EMT) [48–50]⁠, and promotes angiogenesis [51, 52]⁠ and metastasis [43, 45, 53]⁠, which has positioned lactadherin as an interesting possible prognostic biomarker and therapeutic target for BC. However, the mechanisms by which it exerts these pro-tumorigenic or pro-metastatic effects have not been completely elucidated.

It is well known that lactadherin is present in EVs secreted by different cells and in different physiological and pathological contexts [54–56]⁠. However, the presence of lactadherin in BC cell-secreted sEVs and its role in sEV-mediated cellular communication have not yet been described. To better understand the role of lactadherin in BC tumors, we first analyzed TCGA transcriptomic and proteomic data from BC patients to evaluate lactadherin expression levels and its association with survival, tumor stage, and subtype. We also evaluated the presence of lactadherin in metastatic and non-metastatic human BC cell lines. To understand the role of lactadherin in BC cell-secreted sEV-mediated promotion of tumorigenic and metastatic capacities, we evaluated the migration, tumoroid formation, and anchorage-independent growth capacity of non-metastatic recipient cells incubated with sEV-MDA231 after blocking lactadherin in those sEVs. Finally, we evaluated the effects of lactadherin blockade in sEV-MDA231 in the formation of malignant ascites and tumor nodules in a murine peritoneal carcinomatosis model. The results of this study provide the first insights into the role of lactadherin in metastatic BC cell-secreted sEVs as inductors of metastatic capacity in less aggressive recipient cells in vitro and in vivo in a peritoneal carcinomatosis mouse model, supporting the possibility of using lactadherin in sEVs as a new therapeutic agent for this disease.

## Methods

### BC patients TCGA data

Protein and mRNA transcriptomic data from BRCA CPTAC (N = 100) and RNA-seq (N = 1090) datasets were downloaded from the TCGA database in February 2021, using TCGA-Assembler v2 and TCGA-biolinks, respectively [57, 58]⁠. CPTAC protein expression levels were quantile-normalized. Upper-quartile normalized FPKMs were downloaded directly from GDC using TCGA-biolinks and were used for RNA-seq analysis. Group comparisons between stages I-II vs III-IV were performed using the Wilcoxon Rank Sum Test (Mann–Whitney U-test). Comparisons between PAM50 tumor subtypes were performed using the Kruskal–Wallis test, followed by pairwise comparisons using the Wilcoxon Rank Sum Test. P-value adjustment was performed using the Holm’s method. Harmonized survival data from Liu et al. [59]⁠ was used to perform univariate survival analysis between advanced (stages III-IV) and early (stages I-II) BC patients, using progression-free interval as primary outcome, as recommended by TCGA. A sub-analysis of advanced and early BC according to the PAM50 subtype was also performed. Samples were labeled as lactadherin-high and low using a median cutoff value. All survival analyses were conducted using RNA-seq UQ-FPKM values through the implementation of the survival and survminer R packages. In addition, BC patient data and BC cell line data obtained from the UCSC Xena database (downloaded on September 17th, 2020) were used to analyze *MFGE8* (lactadherin coding-gene) levels and their association with ER, PR, and HER2 histologic subtypes.

### Cell lines and cell culture

Human BC cell lines MCF7, T47D, ZR75, and MDA-MB-231 were obtained from ATCC (ATCC^®^ HTB-22TM, ATCC^®^ HTB133TM, ATCC^®^ CRL1500TM, and ATCC^®^ HTB-26TM, respectively) and cultured in DMEM/F12 medium (Gibco) supplemented with 10% fetal bovine serum (FBS; Biological Industries), 100 IU/mL penicillin, 0.1 mg/mL streptomycin and 0.05 mg/mL gentamicin (Gibco). The MCF10A immortalized normal mammary epithelial cell line was kindly donated by Dr. Flavia Bruna (Instituto de Medicina y Biología Experimental de Cuyo (IMBECU), CONICET, CCT-Mendoza, Argentina) and cultured in DMEM/F12 medium supplemented with Brain Pituitary Extract, hEGF, Insulin, Hydrocortisone, and GA-1000 according to the manufacturer’s instructions (Lonza/Clonetics Corporation). All cell lines were cultured at 37 °C and 5% CO_2_. For sEVs isolation, 1.5–2.0 × 10^6^ cells (cell number depends on the cell line used to isolate the sEVs) were seeded in complete medium (supplemented with FBS and antibiotics). Twenty-four hours later, the cell culture medium was discarded, cells were washed twice with sterile and filtered PBS, replaced with 10 mL Optimem (Gibco) per 100 mm2 plate, and supplemented with antibiotics as described. The cell culture supernatant was collected 48 h later to isolate sEVs.

### Antibodies

Antibodies against human lactadherin (monoclonal sc-8029), CD63 (sc-5275 or ab68418), TSG101 (sc-7964), calnexin (sc-46669), GAPDH (sc-47724) and β-actin (sc-47778) were purchased from Santa Cruz Biotechnology, Inc. or Abcam. A policlonal antibody against lactadherin was also used (AF2767-SP) and purchased from RyD Systems. Antibody against human flotillin 1 (18634S) was purchased from Cell Signaling Technology (Danvers, MA). Alexa Fluor 488-conjugated anti-lactadherin antibody (sc-8029) was also purchased from Santa Cruz Biotechnology Inc. Anti-rabbit IgG-HRP (7074S) and anti-mouse IgG-HRP (7076S) secondary antibodies were purchased from Cell Signaling Technology. Goat anti-rabbit IgG-800CW (#92,532,211) and goat anti-mouse IgG-680LT (#925–68,020) secondary antibodies were obtained from Licor.

### qRT-PCR

Total RNA from MCF10A, MCF7, T47D, ZR75, and MDA-MB-231 cell lysates was extracted using TRIzol (Qiagen) following the manufacturer’s instructions. 1 µg total RNA were treated with 1 µL (1 IU) of DNAse I (Invitrogen) for 15 min at 25 °C. The reaction was stopped with 1 µL of EDTA and incubated for 10 min at 65 °C. DNAse-treated RNA was incubated with oligo-dT and reverse-transcribed using MMLV/Superscript RT (Invitrogen) according to the manufacturer’s instructions in a final volume of 20 µL. For lactadherin mRNA (*MFGE8*) detection, the cDNA was diluted with the same volume of nuclease-free H2O and 1–2 μl of diluted cDNA was used for amplification by PCR using a Roche kit (Roche, #12,239,264,001) following the manufacturer’s instructions. The qPCR program used was as follows: denaturation at 95 °C for 10 min and amplification at 95 °C for 10 s, 59 °C for 10 s, and 72 °C for 6 s (45 cycles). The 2–ΔΔCt method was used to calculate the relative levels of *MGFE8* mRNA, and the data were normalized to GAPDH levels. The primer sequences for lactadherin amplification were5′-CCTGCCACAACGGTGGTTTAT-3′ (forward) and 5′-CACATTTCGTCTCACAGTGGTT-3′ (reverse), and for GAPDH, 5′-CTGGGCTACACTGAGCACC-3′ (forward) and 5′-AAGTGGTCGTTGAGGGCAATG-3.’

### Western blot

BC cell monolayers were washed twice with cold PBS and lysed with RIPA buffer (Thermo Scientific). 100X proteases inhibitors cocktail (Pierce, Thermo Fisher Scientific) was then added to the sample. The samples were then sonicated twice in a bath sonicator for 10 min and centrifuged (14,000 × g for 15 min at 4 °C) to obtain protein extracts. Total protein extracts (30–50 µg/lane) were loaded onto 10–12% SDS–polyacrylamide gels and transferred to nitrocellulose membranes. Membranes were blocked (Intercept TBS blocking buffer; Licor) during one hour and probed with primary antibodies against lactadherin (Santa Cruz; 1:400 dilution) and β-actin or GAPDH (Santa Cruz; 1:1000 dilution). Finally, bound antibodies were detected with anti-IgG 800 GW secondary antibodies (Licor; 1:10,000 dilution) and revealed using an Odyssey Clx imaging system (Licor).

### Immunofluorescence detection of lactadherin in BC cell lines

BC cells (2.5 × 10^5^ BC cells were seeded on coverslips in 24 well plates. Twenty-four hours later, coverslips were collected, and cells were fixed with 4% paraformaldehyde (PFA) for 10 min at room temperature (17–23 °C). The coverslips were washed twice with TBS, blocked with TBS/FBS 2% buffer for 30 min at room temperature, and incubated with anti-lactadherin primary antibody overnight at 4 °C. Finally, the coverslips were washed with TBS supplemented with 0.1% Tween and incubated with an anti-IgG Alexa Fluor 488 secondary antibody (Molecular Probes) for 2 h at room temperature. Cells were stained with DAPI (Thermo Fisher Scientific), mounted, and visualized using a confocal microscope (Fluoview FV10i; Olympus).

### Flow cytometry

For surface staining, 2.5 × 10^5^ BC cells were detached, washed with PBS and pelleted by centrifugation at 400 × g for 5 min. The cell pellets were suspended in PBS supplemented with 2% FBS and stained with Alexa Fluor 488-conjugated anti-lactadherin antibody for 30 min in the dark. The cells were washed twice with PBS and 2% FBS and suspended in the same buffer. For intracellular staining, the fixation/permeabilization kit protocol was used, according to the manufacturer’s instructions (eBiosciences, cat 88–8824). Briefly, after detachment, the cells were fixed with 100 µL fixation buffer for 40 min. The cells were washed with permeabilization buffer diluted 1:10 with permeabilization diluent and stained with Alexa Fluor 488-conjugated anti-lactadherin antibody (suspended in permeabilization buffer) for 30 min in the dark. The cells were then washed twice and suspended in PBS plus 2% FBS. In both cases, 1 µL of 7-AAD (Sigma-Aldrich) was used to discard dead cells before acquisition. The cells were analyzed using a Dako Cytomation CyAn ADP flow cytometer. FACS analysis was performed using the FlowJo v10.

### sEVs isolation from cell culture supernatants

1.5–2.0 × 10^6^ BC cells (cell number depending on the cell line used to isolate the sEVs) were seeded in 100 mm plates in complete DMEM/F12 medium. The following day, the cell medium was discarded and replaced with 10 mL/plate Optimem medium (Gibco). Forty-eight hours later (approximately 80% confluence), the cell culture supernatant (Optimem) was collected. Cell culture supernatants were centrifuged first at 3000 × g for 10 min to eliminate cell debris, and then at 10,000 × g for 15 min to eliminate larger vesicles (apoptotic bodies). The supernatants were then filtered through 0.22 µm filters and concentrated using Amicon 100 MWCO tubes (Merck Millipore). Finally, sEVs were obtained through precipitation and size-exclusion columns using the Exo-Spin exosome purification kit, according to the manufacturer’s instructions (Cell Guidance).

### Nanotracking analysis (NTA)

The sEVs were characterized by NTA. Briefly, sEVs isolated from cell culture supernatant isolated were diluted 1:100 with filtered sterile PBS and injected into Nanosight NS300 instrument (NanoSight NTA 2.3 Nanoparticle Tracking and Analysis Release Version Build 0033; Malvern Instruments; Fondequip EQM160157) to measure concentration and size distribution of particles. The camera was set up to capture three videos of 30 s each per sample. The videos were then analyzed to determine the size distribution with an approximation of the quantity of particles (Camera level 11–12; Gain 5).

### Transmission electron microscopy

The sEVs were deposited on Formvar-carbon-coated grids (TED Pella, Mountain Lake, CA, USA). After 1 min of adsorption, the excess was removed with absorbent paper and contrasted with uranyl acetate pH 7.0 for 1 min; the excess was removed and dried for 1–2 min at room temperature. The specimens were observed using a Talos F200C G2 electron microscope at 80 kV (Unidad de Microscopía Avanzada UC (UMA), Pontificia Universidad Católica de Chile, Santiago, Chile).

### sEVs characterization by western blot

For WB analysis, total protein extracts were lysed with RIPA buffer and sonicated twice in a bath sonicator for 10 min. Total protein extracts were mixed with 4X Laemmli loading buffer (Biorad—161–0747), heated at 95 °C for 5 min, and loaded onto 10–12% SDS–polyacrylamide gels (15–20 µg/lane). Proteins were transferred to nitrocellulose membranes that were blocked (Intercept TBS blocking buffer; Licor) during one hour and probed with antibodies against exosome markers (CD63, TSG-101) and negative markers (calnexin) to evaluate possible cellular contamination. Finally, bound antibodies were detected with anti-IgG 680/800 GW secondary antibodies (Licor 1:10,000) and revealed using an Odyssey Clx imaging system (Licor).

### ELISA

To determine the presence of lactadherin in BC cell-secreted sEVs, we used enzyme-linked immunosorbent assay (ab213810; Abcam) according to the manufacturer’s instructions. Briefly, sEVs were prepared as described previously, and 50 µL of each sample was seeded directly into each well. Notably, the sEVs samples were not sonicated previously. One hundred microliters of each sample or standard dilution were added to each well in duplicate. The optical density (absorbance) was read at 450 nm in a microplate reader (within 30 min after adding TMB Stop Solution).

### Transwell migration assay

Boyden chambers were hydrated with DMEM/F12 for 1 h. Cells (2.5 × 10^4^) were seeded in the upper chamber of 24-well transwell plates. Next, ~ 5000 particles per seeded cell (generally 5 µg total protein) and/or 2 μg anti-lactadherin antibody were added to the cells to evaluate their effect on the 3D migration capacity of BC cells. After 16 h, the transwell inserts and membranes were collected, fixed with 4% PFA, and stained with DAPI. Cells on the other side of the inserts were visualized using an inverted fluorescence microscope and counted in at least ten fields.

### Tumoroid formation assay

BC cells (5.0 × 10^5^ MCF10A, 7.5 × 10^4^ MCF7, or MDA-MB-231) were seeded on sterile 2% agar-covered plates (6 well-plates), supplemented with Mammary Epithelial Cell Growth culture medium (at least 1.5 mL of MEGM™ (Lonza, cat. CC-3151) supplemented with EGF 25 ng/mL, hydrocortisone 0.5 g/mL, insulin 5 µg/mL (Lonza, cat. CC-4136) and bFGF 25 ng /mL (Invitrogen, cat PHG0026). Tumoroids (tumor spheroids) were grown at 37 °C and 5% CO_2_ for 14 days. The cell culture medium was not renewed during the 14 days of the experiment, and the formation of spheres was visually recorded by photography using the Micrometrics SE Premium 4 software in a Nikon Eclipse TS100 inverted microscope. After 14 days, the cell culture medium with the spheres was extracted from the well and passed through a 70 µm filter (BD Falcon, cat.352350). The spheres retained on the filter were recovered and plated on a 12-well adhesion plate (Corning, cat. 3512). After 24 h, the adhered spheres were fixed with 4% PFA in 1X PBS for 10 min, washed, and stained with DAPI 1:300 for 10 min. Finally, the spheres were washed 3 times with 1X PBS and visualized and recorded under the same microscope to confirm that the recovered spheres remained viable.

### Anchorage-independent growth evaluation. Colony formation

Anchorage-independent cell growth capacity was determined by colony formation on soft agar as previously described [26, 45]⁠. Briefly, MCF10A, MCF7, T47D, ZR75, and MDA-MB-231 (5.0 × 10^3^, 7.5 × 10^3^, 5.0 × 10^3^, 5.0 × 10^3^, and 3.5 × 10^3^, respectively) cells were seeded in 12-well plates, suspended in soft agar, and incubated for 24 h. After that, the cells were treated with 10 μg of total protein sEVs (equivalent to 5000 particles/per cell), 2 μg of anti-lactadherin antibody, or left untreated. Formation of colonies > 100 μm in diameter was scored 1 and 2 weeks after treatment under a phase-contrast microscope at 10X as described [26, 45]⁠. At least ten fields per condition were counted.

### Animals

Animal studies were conducted in accordance with the guidelines of the Ethical Committee of Clínica Alemana Universidad del Desarrollo. Immune-compromised NOD/SCID mice were obtained from Jackson Laboratories (Bar Harbor, ME). Mice were maintained in the animal facility of Facultad de Medicina, Clínica Alemana Universidad del Desarrollo, in an exclusive, temperature-controlled environment, conditioned with HEPA filters, with a 12/12 h light/dark schedule, and sterile food and water ad libitum.

### Peritoneal carcinomatosis assay

Four groups of seven NOD/SCID mice (two separate experiments) were inoculated intraperitoneally (ip) with 2.0 × 10^6^ MDA-MB-231 cells in 200 μL saline, either alone (saline group) or together with sEV-MDA231, anti-lactadherin antibody, or sEV-MDA231 + anti-lactadherin antibody (10 μg sEV-MDA231 + 2 μg antibody per mouse) (1st dose). Seven days after tumor cell inoculation, the mice were treated again with sEV-MDA231 or anti-lactadherin antibody (2nd dose). The mice were treated on days 7, 9, 11, 15, 17, and 19 (six doses). The animals were euthanized 21 days after cell injection. The mesenteric tissue and retroperitoneal tumor masses were excised and fixed in 4% PFA. Malignant ascites were collected, and the total cell number was determined by trypan blue exclusion assay. For tumor micronodule measurements, the tumor tissue were fixed and paraffin-embedded. Histological sections of mesenteric tissue were first stained with hematoxylin and eosin (HE). Images of the HE-stained sections were gray-scale-transformed to define a hyperdense area with ImageJ software. Hypercromic hematoxylin + areas corresponded to tumor micronodules (originally in purple; now delimited in white). The number of tumor micronodules in the mesenteric tissues of the peritoneal cavity were quantified, marked and manually delimited by hyperchromia. Tumor area was measured in ImageJ software by gray range density/intensity, which coincides with denser nuclear staining of tumor cells relative to total area. Additionally, measurements of tumor area (relative to total tissue area), can be calculated using mathworks software (https://es.mathworks.com). At least 10 histological sections were analyzed in a double-blind manner by an expert pathologist from Hospital Luis Tizné (Santiago, Chile).

### Statistical analysis

Graphs and statistical analyses were performed using GraphPad Prism v8 software. A non-parametric Mann–Whitney U test was used to compare the two groups. Two-tailed parametric ANOVA or non-parametric Kruskal–Wallis test was used to compare three or more groups. Tukey’s or Dunn’s correction was used for multiple comparisons as correspond. Differences were considered statistically significant at P < 0.05.

## Results

### MFGE8 transcription is associated with worse survival in BC patients

**Fig. 1:**
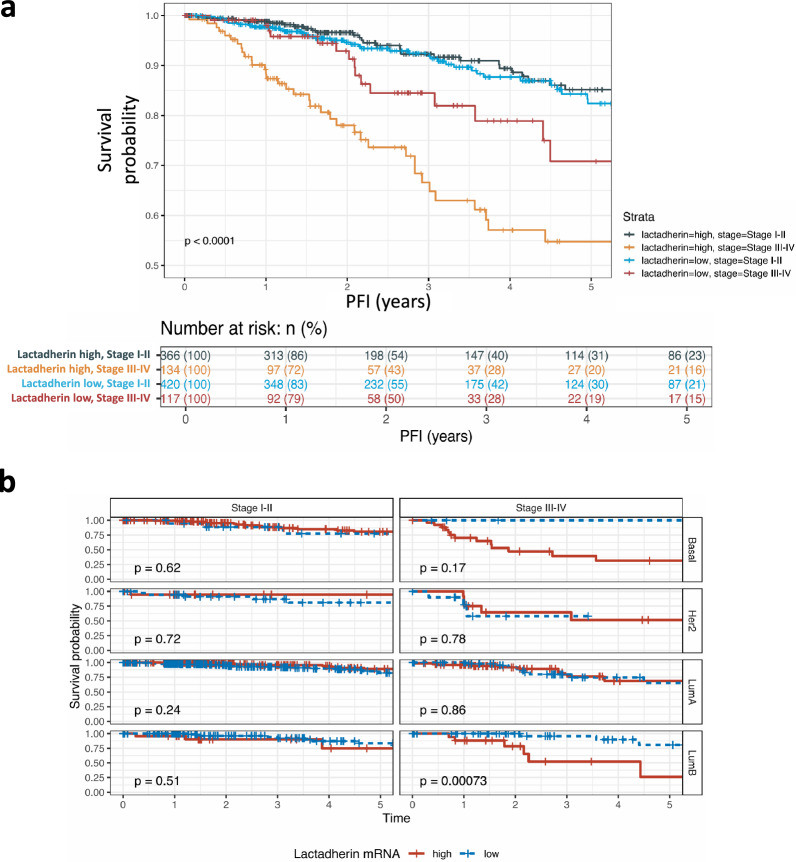
*MFGE8* expression is associated with worse survival in BC advanced stages and more aggressive PAM50 subtypes.** A** Harmonized survival data from Liu et al. [59]⁠ was used to perform univariate survival analysis between advanced (stages III-IV) and early (stages I-II) BC patients, using progression-free interval as primary outcome, as recommended by TCGA. **B** Sub-analysis between advanced and early BC by PAM50 subtype was also performed. Samples were labeled as *MFGE8*-high and low using a median cutoff value. All survival analyses were conducted using RNA-seq UQ-FPKM values, through implementation of the survival and survminer R packages

To evaluate the significance of lactadherin transcription in patients with BC, we performed survival analysis by subdividing patients with low and high *MFGE8* levels. BC patients in the early stages of the disease had similar survival probabilities, independent of *MFGE8* levels (blue and light blue curves). On the other hand, patients with higher tumor stages (III and IV) and high *MFGE8* transcription had worse survival than patients with low *MFGE8* transcription (brown and orange curves) (Fig. 1A). Moreover, *MFGE8* transcription was also associated with worse survival in BC patients with more aggressive PAM50 subtypes, such as luminal B (p = 0.00073), basal (although not statistically significant; p = 0.17), and normal-like subtypes (not shown), which were also associated with advanced stages of the disease (Fig. 1B).

### Lactadherin transcription (mRNA) and expression (protein) are associated with more aggressive subtypes in BC patients and cell lines

**Fig. 2:**
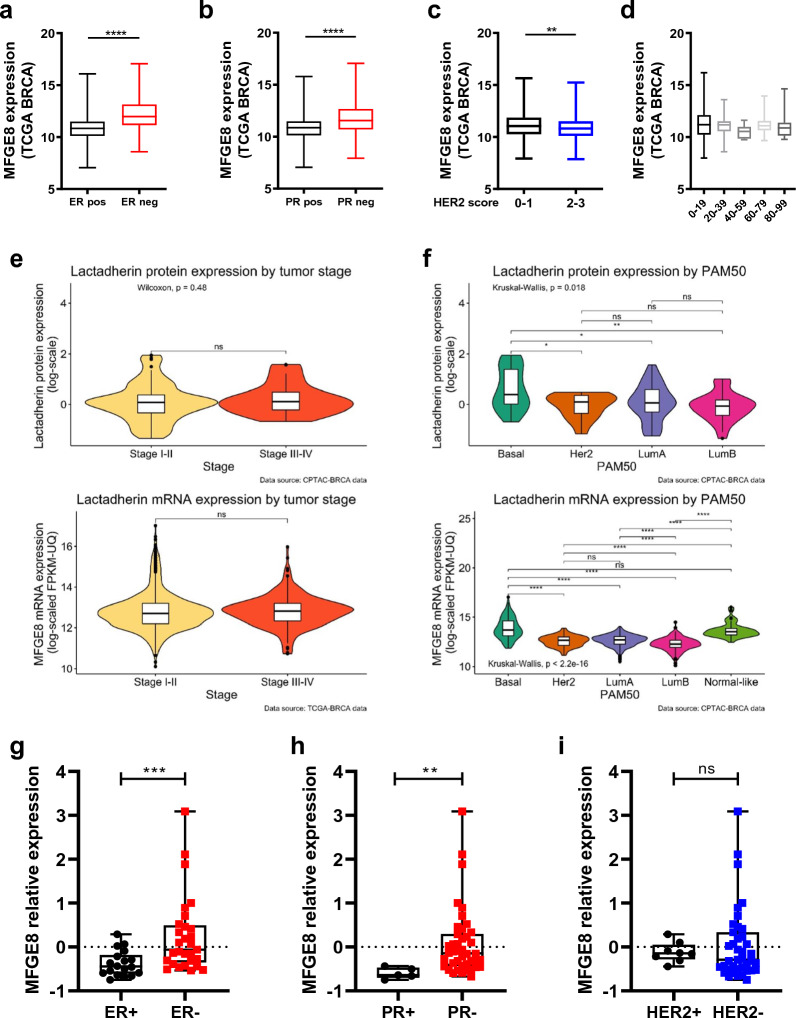
Lactadherin levels are associated with more aggressive BC subtypes. Analysis of TCGA data show that *MFGE8* expression is higher in ER- and PR- BC patients and in more aggressive basal and normal-like PAM50 subtypes. **A-D** BC patients’ TCGA transcriptomic data were obtained from UCSC Xena database and *MFGE8* expression were associated with ER, PR and HER2 histologic status. **E–F** Transcriptomic and proteomic data from another cohort of BC patients were downloaded directly from TCGA and association analyses of lactadherin levels with tumor stage **E** and PAM50 subtypes were performed **F**. **G-I** TCGA transcriptomic data from BC cell lines were obtained from UCSC Xena database and *MFGE8* expression were associated with ER, PR and HER2 histologic status. Mann–Whitney test statistical analysis was performed **A-C**; Kruskall Wallis test and Dunn’s multiple comparisons correction were performed **D**; Group comparisons between stages I-II vs III-IV were carried out using Wilcoxon Rank Sum Test (Mann–Whitney U-test) **E**; Comparisons between PAM50 tumor subtypes were performed with Kruskal–Wallis test, followed by pairwise comparisons using Wilcoxon Rank Sum Test. P-value adjustment was performed using Holm’s method **F**; Kruskal–Wallis test statistical analysis was performed **G-I**

Next, we downloaded and analyzed BC patient and BC cell line data from TCGA directly or the UCSC Xena database to evaluate the association of *MFGE8* mRNA and lactadherin protein levels with histologic BC subtypes, tumor stage, and PAM50 subtypes. First, we observed that *MFGE8* levels were associated with the absence of estrogen and progesterone receptors (higher in ER- and PR- tumors, respectively) (Fig. 2A-B). In addition, *MFGE8* transcription was slightly lower in patients with increased HER2 detection; however, this difference was lost when the patients were subdivided according to HER2 + cell percentage (Fig. 2C-D). Then, using another cohort, we analyzed *MFGE8* transcription and lactadherin protein levels (expression) and their association with tumor stage and the PAM50 subtype. Lactadherin levels were not associated with tumor stage, either at the proteomic or transcriptomic level (Fig. 2E). On the other hand, interestingly, both lactadherin transcription (measured by RNA-seq) and protein expression (measured by mass spectrometry) were increased in basal (protein) and basal and normal-like PAM50 subtypes (mRNA), respectively (Fig. 2F). Furthermore, we observed that *MFGE8* mRNA was increased in ER- and PR-BC cell lines. However, no association was found with HER2 presence (Fig. 2G-I). All these analyses show that the presence of lactadherin, both at the mRNA and protein levels, is associated with more aggressive subtypes in BC patients and cell lines, suggesting that it could be used as a biomarker of worse prognosis and as a therapeutic option in some BC patients.

### Lactadherin is differentially expressed in human BC cell lines

**Fig. 3:**
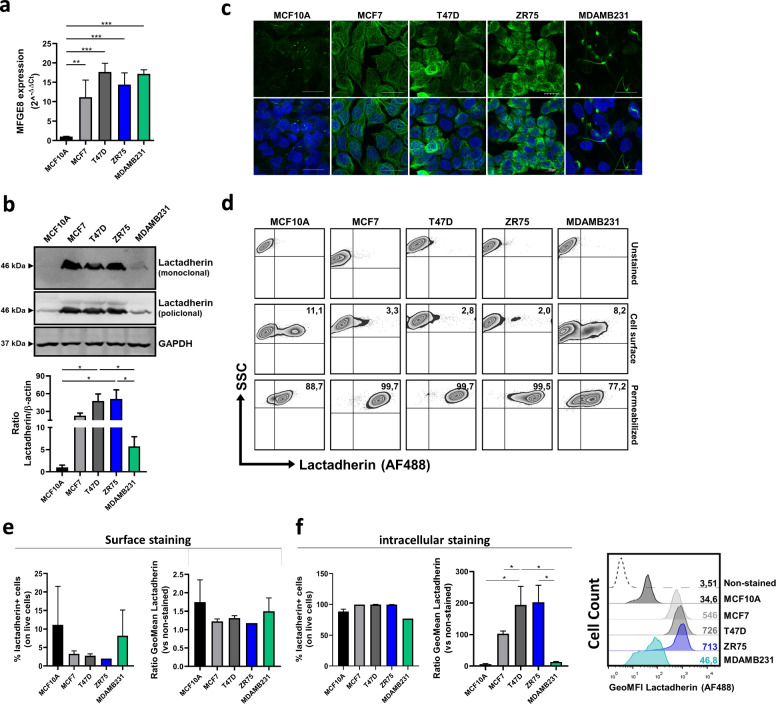
Lactadherin is differentially expressed in human BC cell lines.** A** Lactadherin (*MFGE8*) mRNA expression was measured in different human BC cell lines and in mammary epithelial breast cells through RT-qPCR. Lactadherin (*MFGE8*) mRNA expression was normalized against GAPDH; **B** Lactadherin protein was detected through WB analysis. Data was normalized against GAPDH detection for quantification; **C** Confocal microscopy evaluation of lactadherin in different BC cell lines. 120X, z-stack augmented images are shown. Scale bar represents 20 µm; **D-F** Lactadherin detection in BC cells by flow cytometry. Cell surface **D** and intracellular **E, F** histograms and quantifications are shown. The gating strategy is depicted in Additional file 1: Figure S2. mRNA **A** and protein **B** levels are shown as mean ± SEM. Images and data are representative of 3–4 independent experiments. * p < 0.05; ** p < 0.01; *** p < 0.001

It has been shown that lactadherin is differentially expressed in several BC cell lines correlating with their aggressiveness. We used four different human BC cell lines, each with different tumorigenic properties, and one normal human mammary epithelial cell line to assess the mRNA and protein expression of lactadherin. Human BC cell lines MCF7 (ER + , PR + , HER2-), T47D (ER + , PR + , HER2-), ZR75 (ER + , PR-, HER2-), and MDA-MB-231 (ER-, PR-, HER2-) all expressed higher *MFGE8* mRNA levels compared with normal mammary epithelial cells MCF10A (Fig. 3A). Interestingly, there were no differences in *MFGE8* mRNA levels among the different BC cell lines. Rarely, TCGA and DepMap transcriptomic data from these BC cell lines showed higher *MFGE8* transcription in MDA-MB-231 TNBC cells than in any of the other BC cells (Additional file 1: Figure S1), which agrees with the existing literature. In contrast, WB analysis showed that lactadherin protein levels were higher in luminal-like MCF7, T47D, and ZR75 cell lines and lower in MDA-MB-231 cells (Additional file 1: Fig. 3B). Moreover, confocal microscopy and flow cytometry analysis showed that the majority of lactadherin protein is located intracellularly and not on the cell surface of BC cells. Again, as we saw in the WB analysis, lactadherin levels were significantly higher in luminal-like MCF7, T47D, and ZR75 BC cells than in triple-negative MDA-MB-231 and non-tumorigenic MCF10A cells, being expressed mainly intracellularly (Fig. 3C-F). In particular, we saw that surface staining with anti lactadherin antibody only stained between 2.0–11.1% of tumor cells; this percentage being higher in MCF10A normal mammary epithelial cells and in MDA-MB-232 TNBC cells (Fig. 3D). In contrast, when performing intracellular staining, between 77.2–99.7% were positive for lactadherin (Fig. 3E), however, it is clear that its expression (expressed as GeoMFI) is higher in MCF7, T47D and ZR75 luminal-like BC cells (Fig. 3F).

### Lactadherin protein is present in BC cell-secreted sEVs

**Fig. 4:**
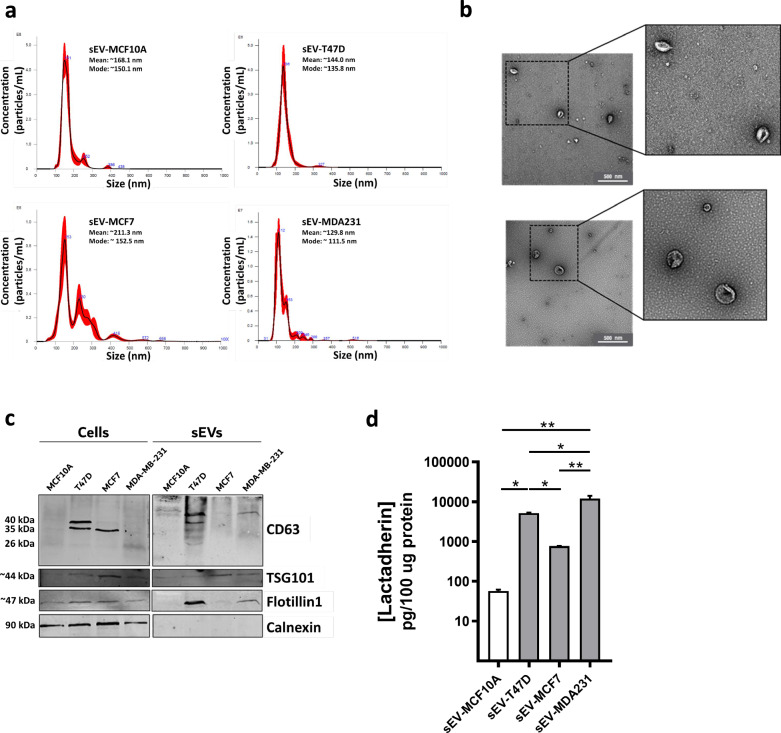
Lactadherin protein is present in BC cell-secreted sEVs. sEVs secreted by different human BC cell lines were obtained through cell culture conditioned medium ultracentrifugation and the number, size and integrity were analyzed by **A** Nanotracking analysis and **B** Transmission electron microscopy (representative images of sEV-MCF10A (up) and sEV-MDA231 (down)). **C** Detection of sEV markers in human BC cell-secreted sEVs. Total protein extracts were loaded in 10–12% acrylamide gels and transferred to nitrocellulose membranes. CD63, TSG101 and Flotillin-1 were used as sEV markers. Calnexin was used as intracellular contamination protein. Human normal mammary epithelial MCF10A cells was used as control. **D** Lactadherin protein level was measured in sEVs through an ELISA assay. Fifty microliters of each sEV per well were used in the assay. Results were normalized against 100 µg total protein amount, quantified by microBCA total protein quantification assay. The graph shows the mean ± SD. Images and data are representative of three independent experiments. * p < 0.05; ** p < 0.01

sEVs are important players in intercellular communication and are implicated in tumorigenesis and metastasis [8–11, 13, 14, 16]. On the other hand, lactadherin overexpression has been associated with poor prognosis in several types of cancers, including BC [60–62]⁠. Thus, we evaluated the presence of this protein in BC cell-secreted sEVs obtained from MCF7, T47D, and MDA-MB-231 BC cell supernatants using ultracentrifugation (sEV-MCF7, sEV-T47D, and sEV-MDA231). We also obtained sEVs from MCF10A normal (non-tumorigenic) mammary cells as controls (sEV-MCF10A). Nanotracking analysis (NTA) showed that BC cell lines secreted more sEVs than normal mammary MCF10A cells (Additional file 1: Figure S3A). Both the mean size and mode size of sEVs seemed to be similar in all isolated sEVs (Additional file 1: Figure S3B–C). In addition, their size distributions were similar to those evaluated by NTA and transmission electron microscopy (TEM) (Fig. 4A-B, Additional file 1: Figure S3D). However, the exception seems to be the smaller fraction of sEVs (0–100 nm), which was higher in sEV-MDA231 (~ 50%) than in the other sEVs (Additional file 1: Figure S3D). All sEVs contained typical sEV markers such as CD63 and TSG101 (and Flotillin-1 in two out of four sEVs) and did not express the cellular marker calnexin (Fig. 4C), thus showing no contamination with cellular components. Interestingly, ELISA showed that lactadherin was almost undetectable in sEV-MCF10A but was present in sEVs secreted by all BC cell lines. Moreover, lactadherin levels in those sEVs was associated with BC cell line aggressiveness, being higher in sEV-MDA231 than in sEV-MCF7 and sEV-T47D (Fig. 4D), suggesting a possible role of lactadherin in sEV-mediated cellular communication.

### Lactadherin blockade in sEV inhibits sEV-MDA231-mediated increase of the migration capacity less aggressive non-metastatic BC recipient cells

**Fig. 5:**
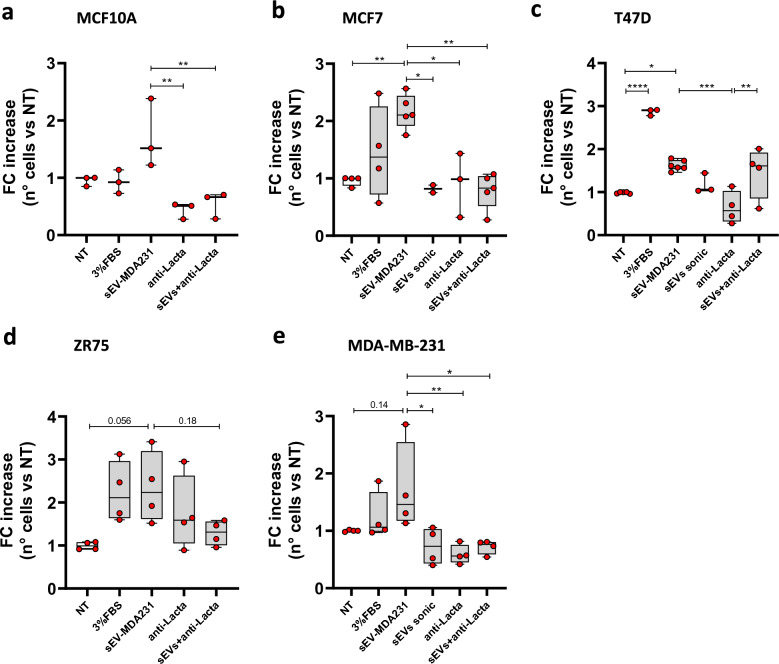
Blockade of lactadherin in sEV-MDA231 inhibits their pro-migration effect on recipient cells. Different human BC cells were incubated with metastatic sEV-MDA-MB-231 pre-treated or not with a lactadherin blocking monoclonal antibody to evaluate its role in their migratory capacity by a transwell migration assay. **A** MCF10A, **B** MCF7, **C** T47D, **D** ZR75 and **E** MDA-MB-231 recipient cells were seeded on PET-membrane Boyden transwell chambers and treated once with sEV-MDA231 (blocked or not with anti-lactadherin antibody for 16 h. After that, membranes were collected, fixed with 4% PFA and stained with DAPI. Cells into the membrane were counted in 10 fields on an inverted fluorescence microscope and then quantified. Graphs show mean ± SEM. * p < 0.05; ** p < 0.01; *** p < 0.001; **** p < 0.0001

It has been shown that sEVs secreted by metastatic BC cells (i.e. MDA-MB-231) can induce changes in less aggressive, non-metastatic recipient cells, increasing tumorigenic and metastatic properties such as migration and invasion [12, 26, 37, 64–66]. However, there are no reports implicating lactadherin in these sEV-mediated changes. To assess whether sEV-MDA231 promotes tumorigenic or metastatic changes in other BC recipient cells, and to evaluate the role/contribution of lactadherin present in sEV-MDA231, non-metastatic MCF7, T47D, or ZR75, and non-tumorigenic MCF10A cells were incubated with sEV-MDA231 cells that were previously treated with a blocking monoclonal antibody against lactadherin, and the migratory capacity of the recipient cells was evaluated. As expected, non-treated/blocked sEV-MDA231 induced a 1.71-, 2.16-, 1.62-, 2.35-, and 1.73-fold increase in MCF10A, MCF7, T47D, ZR75 and MDA-MB-231 3D migration capacity, respectively (Fig. 5A-E). Interestingly, previous lactadherin blockade on sEV-MDA231 suppressed their pro-migration effect. However, unlike what was reported in other studies, anti-lactadherin antibody alone did not seem to significantly decrease basal migratory levels (NT vs. anti-Lacta comparison), suggesting that the blocking of lactadherin present in sEV-MDA231 is a direct effect on sEVs and does not target membrane-associated cellular lactadherin.

### Lactadherin blockade in sEV inhibits sEV-MDA231-mediated increase of the spheroid/tumoroid formation capacity of recipient cells, but not their anchorage-independent growth

**Fig. 6:**
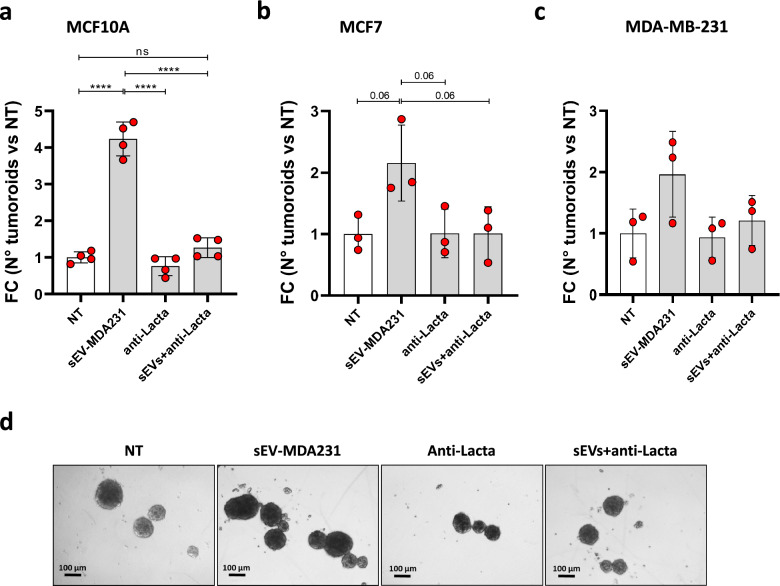
Blockade of lactadherin in sEV-MDA231 inhibits their pro-tumoroid formation potential. Different human BC cells were incubated with metastatic sEV-MDA231 pre-treated or not with a lactadherin blocking monoclonal antibody to evaluate its role in promoting spheroid/tumoroid formation. After 14 days, spheroids/tumoroids were visualized and photographed under an inverted microscope. Spheroids/tumoroids with a size ≥ 100 µm were quantified **A**. Representative images of spheroids/tumoroids formed in the different conditions after 14 days are shown **D**. The size bar corresponds to 100 µm. All data is representative of 3 independent experiments. **** p < 0.0001

**Fig. 7:**
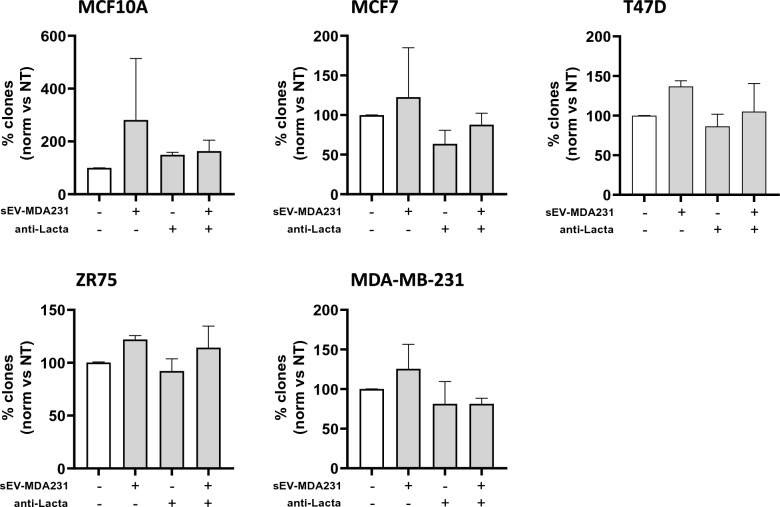
sEV-MDA231 does not promote anchorage-independent growth of recipient BC cells. Anchorage-independent growth (clonogenic assay) was evaluated in recipient cells incubated with sEV-MDA231 previously treated or not with a lactadherin blocking antibody. 3.0–7.5 × 10^3^ recipient cells were seeded in semisolid cell culture medium. Next day cells were treated with sEV-MDA231 (and every other condition day 1 (D1)). Another supplement was added at day 8 (D8). The number of clones formed were evaluated at day 14 (D14), visualized under inverted optic microscope. Each condition was tested in duplicates and at least 10 fields per condition were evaluated. Graph shows mean ± SEM. All data is representative of 3 independent experiments

Additionally, we evaluated the capacity of sEV-MDA231 to increase the tumoroid formation capacity and anchorage-independent growth (clonogenic capacity) of recipient cells, which are directly related to stem cell capacities and the potential of tumor cells to survive in circulation [67, 68]. At the same time, we aimed to evaluate the inhibitory effect of lactadherin blockade in those sEV-MDA231. sEV-MDA231 promoted the tumoroid formation potential of less aggressive recipient cells, increasing the number of viable spheroids formed (Fig. 6, Additional file 1: Figure S4). Previous lactadherin blockade of sEV abrogated their pro-tumoroid effect. Again, anti-lactadherin antibody treatment alone did not decrease the basal tumoroid formation capacity of the treated cells. Of note, the effect of sEV-MDA231 was more evident, promoting spheroid formation of normal mammary epithelial cells such as MFC10A cells (Fig. 6A); however, despite the fact that they slightly increased tumoroid formation of MCF7 and MDA-MB-231 BC cells, these increases were not statistically significant (Fig. 6B-D). On the other hand, with our experimental setting/approach, neither sEV-MDA231 alone, anti-lactadherin alone, nor the combination of treatments had any effect on the anchorage-independent growth capacity of recipient/treated cells (Fig. 7), suggesting that the cargo or the dose of sEV-MDA231 may not be sufficient to promote this particular capacity. Taken together, these results suggest an important role for lactadherin present in sEV-MDA231 mediating some tumorigenic and metastatic effects of these nanovesicles.

### *Lactadherin blockade inhibits sEV-MDA231-mediated ascites and tumor micronodules formation in an *in vivo* peritoneal carcinomatosis metastatic murine model*

**Fig. 8:**
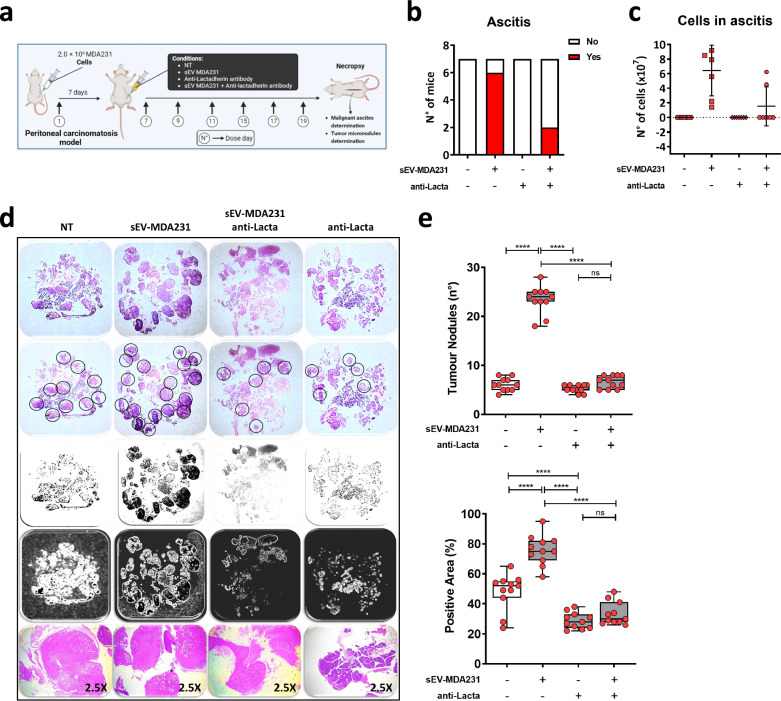
Lactadherin blockade inhibits sEV-MDA231-mediated ascites formation and mesenteric/peritoneal tumor micronodules formation in an in vivo peritoneal carcinomatosis murine model. NOD/SCID mice (two separated experiments) were inoculated intraperitoneally (ip) with 2.0 × 10^6^ MDA-MB-231, either alone (saline group) or together with sEV-MDA231, anti-lactadherin antibody or sEV-MDA231 + anti-lactadherin antibody. Seven days after tumor cells inoculation, mice were treated again on days 7, 9, 11, 15, 17 and 19 (7 total doses). Mice were euthanized on day 21 post-cell injection and organs were collected in the necropsy (**A**). When present, malignant ascites were also collected and total cell number was determined by trypan blue exclusion assay (**B-C**). For tumor micronodules measurement, tumor tissues were fixed and paraffin-embedded. Histologic sections of mesenteric tissue were first stained with HE. Images of HE-stained sections were gray scale-transformed to define a hyperdense area. Tumor micronodules number in mesenteric tissues of the peritoneal cavity were evaluated by purple hyperchromia. Tumor nodules and tumor area were also measured by gray range density/intensity, which coincides with the denser nuclear staining of tumor cells (**D-E**), and the quantified. Analyses were made using ImageJ software and corroborated using mathworks software (https://es.mathworks.com). **E** Tumor nodules number (upper graph) and area (lower graph) are shown. The final in vivo evaluation was performed in a double-blinded fashion and corroborated by anatomopathological analysis

Finally, to evaluate whether lactadherin present in sEV-MDA231 plays a role in promoting metastasis in vivo, we used a peritoneal carcinomatosis mouse model in which we injected tumor cells directly into the peritoneal cavity of mice. We injected 2.0 × 10^6^ highly metastatic MDA-MB-231 breast tumor cells on day 0 and, on the same day, treated mice with either sEV-MDA231 (10 µg total protein) or lactadherin-blocked sEV-MDA231 (Fig. 8A). With this model, we can evaluate metastasis by directly detecting tumor growth in retroperitoneal organs such as the spleen, liver, kidneys, and mesentery, and evaluate the formation of malignant ascites and the number of tumor nodules formed. We observed that treatment with sEV-MDA231 increased tumor growth. The total tumor mass was higher in mice treated with sEV-MDA231, an increment that was partially reversed (not statistically significant) when lactadherin in the sEVs was previously blocked (Additional file 1: Figure S5A). In addition, sEV-MDA231 treatment did not change the liver, kidney, or lung size (Additional file 1: Figure S5B-D). However, sEV-MDA231 treatment significantly promoted the formation of malignant ascites in the peritoneal cavity of mice; 86% of mice treated with sEV-MDA231 developed malignant ascites. Interestingly, this effect was drastically inhibited when sEV-MDA231 were previously treated with anti-lactadherin antibody (Fig. 8B, C), suggesting an important role of lactadherin in sEV-MDA231-mediated ascites formation. Notably, in this in vivo model, malignant ascites formation was completely abrogated in mice treated with the anti-lactadherin antibody alone. On the other hand, mice that received sEV-MDA231 developed more and larger tumor nodules than untreated mice. In contrast, mice treated with sEV-MDA231 previously blocked with anti-lactadherin antibody or treated with anti-lactadherin antibody alone developed the same numbers but relatively smaller tumor nodules than non-treated mice (Fig. 8D, E), suggesting that anti-lactadherin treatment and blockade of lactadherin in sEVs play and important role in tumor and ascites formation, in vivo.

## Discussion

It has been shown that lactadherin is associated with poor prognosis and bad outcomes in several types of cancer [43, 60–62]. However, little is known about the role and importance of its presence in sEVs, and whether it can influence sEV-mediated tumorigenic and/or pro-metastatic effects. First studies implicated lactadherin as a breast tumor marker or a possible therapeutic target for this disease [39, 69]. Some reports [47]⁠ and open access data such as that of UCSC Xena and DepMap, have shown that lactadherin mRNA is highly expressed in metastatic and more aggressive BC cell lines. However, few studies have analyzed lactadherin protein levels in BC samples, cell lines, or sEVs. Here, we first categorized TCGA BC transcriptomic data according to lactadherin (*MFGE8*) gene expression levels (median-categorized) in patients with high and low *MFGE8* levels to perform survival analyses in terms of tumor stage and PAM50 BC subtype. We found that BC patients with low tumor stage had similar survival rates independent of *MFGE8* levels. However, interestingly, BC patients with higher tumor stages (III and IV) and high *MFGE8* levels had worse survival than *MFGE8* low patients (Fig. 1A). On the other hand, *MFGE8* high patients had worse survival when they also had more aggressive PAM50 subtypes, such as luminal B and basal-like subtypes (Fig. 1B). According to previous data, we found that *MFGE8* mRNA levels (from transcriptomic TCGA data) were associated with the absence of ER, PR, and HER2 receptors in BC patients (Fig. 2A-C). This association with the presence/absence of HER2 was lost if the patients were subdivided according to the HER2 + cell percentage (Fig. 2D). Moreover, we also analyzed lactadherin proteomic data and found that neither mRNA nor protein levels were associated with tumor stage (Fig. 2E). However, both protein and mRNA levels were associated with more aggressive PAM50 subtypes, such as the basal and normal-like subtypes (Fig. 2F). Furthermore, according to the patients’ transcriptomic data, we observed that *MFGE8* mRNA was higher in ER- and PR-BC cell lines, but was not associated with HER2 expression (Fig. 2G-I). Taken together, despite the fact that proteomic data from BC patients is scarce, and there are few BC patients with these aggressive subtypes, this is the first time that lactadherin mRNA and protein data from BC patients is analyzed together and associated with cancer aggressiveness and worse outcomes (Figs. 1–2). It would be interesting and important that more proteomic studies on BC and other malignancies be carried out and deposited in public databases in order to increase patient samples and robustness of future analyses.

Next, we evaluated lactadherin mRNA and protein expression in different BC cell lines with different intrinsic properties and aggressiveness. Lactadherin (*MFGE8*) mRNA levels measured by qPCR and protein levels evaluated by immunohistochemistry in most cases have been previously associated with aggressiveness in BC patients’ samples and cell lines [reviewed in 40]. Here, we showed that *MFGE8* mRNA level is higher in BC cell lines than in normal mammary epithelial MCF10A cells. Interestingly, in contrast to other studies, we saw that *MFGE8* level was similar in all BC cell lines tested, independent if they are metastatic (triple-negative MDA-MB-231) or non-metastatic (luminal-like MCF7, T47D, and ZR75) BC cell lines (Fig. 3A). On the other hand, at the protein level, we detected lower levels of lactadherin in MCF10A non-tumorigenic cells than in BC cell lines by WB. However, lactadherin protein levels were higher in hormone receptor-positive BC cell lines (luminal-like) than in triple-negative BC cells (basal-like) (Fig. 3B). These results are in contrast with previous reports that showed that lactadherin protein levels are higher in MDA-MB-231 cells and other triple-negative BC cells than in luminal-like BC cell lines, which is associated with BC cell aggressiveness [70]. To better characterize the presence of lactadherin in BC cell lines, we performed immunofluorescence confocal microscopy and FACS analyses. We observed that most of lactadherin protein was located intracellularly and not on the surface of BC cells. As we saw in the WB analysis, lactadherin levels were significantly higher in luminal-like MCF7, T47D, and ZR75 BC cells than in triple-negative MDA-MB-231 and non-tumorigenic MCF10A cells, mainly intracellularly (Fig. 3C, D, E, F).

Next, as sEVs play essential roles in tumor progression and metastasis, we evaluated the presence of lactadherin in sEVs secreted by different BC cell lines. NTA, TEM, and WB analyses showed that sEVs secreted by the different BC cell lines and isolated by ultracentrifugation had size distributions and morphological characteristics of sEVs (Fig. 4A, B, C). Recent literature has shown that lactadherin can be present in sEVs of different origins [54–56, 71–73]. For example, a recent study showed that their presence in sEVs can discriminate between patients with prostate cancer and healthy subjects [55]⁠. Our results also show that lactadherin protein is present in sEVs secreted by these cell lines. Strikingly, despite expressing low cellular protein levels, lactadherin was highly present in sEV-MDA231 compared with sEV-MCF10A (normal mammary), sEV-MCF7, and sEV-T47D (non-tumorigenic) (Fig. 4D), as measured by ELISA. Interestingly, FACS analysis showed that the membrane fraction was higher in normal mammary epithelial cells (MCF10A) and metastatic triple-negative BC cells (MDA-MB-231) than in non-metastatic BC cells (MCF7, T47D, and ZR75), which were mostly intracellular (Fig. 3C). Importantly, sEVs were not disrupted prior to the ELISA assay, indicating that lactadherin sEVs detection should correspond predominantly to the membrane-associated fraction. These findings could explain, to a certain degree, why lactadherin protein is enriched in sEV-MDA231 compared to the other sEVs analyzed (Fig. 4D) and become relevant because it suggests that lactadherin can be blocked with antibodies or specific peptides; thus, it could be used as a target in adjuvant combined BC therapy. Similarly, as sEVs were not disrupted prior to the ELISA, we cannot discard the possibility that a fraction of lactadherin could also be present inside the sEVs. It could also be possible that the secreted fraction of lactadherin was higher in MDA-MB-231 TNBC cells than in the others non-metastatic BC cell lines, and that this explains why we detect lower cellular lactadherin levels in the more aggressive MDA-MB-231 cells (Fig. 3). Additional experiments need to be performed to evaluate this possibility; for instance, evaluating lactadherin protein in BC cells raw secretome (cell culture conditioned medium) or inhibiting the secretory pathway or the exosome/sEVs biogenesis.

To evaluate the effect or role of lactadherin in the sEV-MDA21-mediated promotion of tumorigenic and metastatic capacities in recipient cells, we performed functional assays to evaluate migration, spheroid/tumoroid formation, and anchorage-independent growth capacity of recipient cells treated with sEV-MDA231, previously blocked or not with a monoclonal blocking anti-lactadherin antibody. In this study, we observed that specific blockade of lactadherin present on sEV-MDA231 inhibited sEV-mediated increase in migration and, to some extent, spheroid/tumoroid formation capacities of recipient cells, but had no effect on anchorage-independent growth (Fig. 5, 6, 7). The different outcomes obtained in this later assay could be attributable to our experimental setting/approach, suggesting that the cargo or dose of sEV-MDA231 administered is not sufficient to promote this particular capacity. In this regard, the proteomic cargo of sEV-MDA231 has been characterized in recent studies [74]⁠. More importantly, gene ontology analysis demonstrated the prevalence of particular biological functions and signaling pathways that closely reflect the associated clinical pathophysiology of each cell line. These findings highlight the value of EVs proteomics as a molecular signature for subtyping BC. By performing in silico analysis of available proteomic data [74]⁠, we found that lactadherin is underrepresented in BC cell lines, whereas it is highly enriched in EVs, being most enriched in EVs secreted by TNBC cells (such as MDA-MB-231), followed by EVs secreted by HER2- (ER + PR +) (such as MCF7 or T47D) and HER2 + cells in decreasing order (Additional file 1: Figure S6).

On the other hand, the use of specific blockade of cellular lactadherin has been reported to have antitumor effects in several types of cancer [50, 75, 76], which suggests that the use of lactadherin-blocking agents in preclinical in vivo tumor growth assays could be an excellent focus for immunotherapy [77]⁠. However, contrary to previous reports, the blockade of cellular lactadherin had no effect on the oncogenic capacities that we tested. It is important to mention that, as shown in Fig. 3, the lactadherin protein is located mainly intracellularly; therefore, it makes sense that the use of an antibody extracellularly does not have significant effects. It could also be possible that the specific antibody used in our experiments has less blocking activity in vitro or is not capable of inhibiting oncogenic intracellular signaling pathways; this particular possibility need to be tested in future works. On the other hand, to the best of our knowledge, this is the first time that the blockade of this protein in sEVs has been evaluated to inhibit sEV-mediated tumorigenic or pro-metastatic effects.

Finally, our in vivo peritoneal carcinomatosis model data showed that simultaneous systemic lactadherin blockade (using the same antibody) or lactadherin blockade in sEV-MDA231 previous to their administration had similar effects on the inhibition of mesenteric tumor micronodules and malignant ascites formation (Fig. 8). In this case, anti-lactadherin antibody alone could have an antitumor effect similar to that reported so far, despite the fact that MDA-MB-231 BC cells have a small amount of membrane-associated lactadherin, as shown in Fig. 3. In this regard, it has been reported that lactadherin-deficient mice develop less advanced tumors [43]. Importantly, the apparent discrepancy/incomplete agreement between our in vitro and in vivo results could be explained by the complexity and heterogeneity of living organisms. The highly complex cell communication between different cell types, such as tumor and non-tumor cells, could be essential to elicit different and more complex responses, which may be incomplete when evaluated in vitro, where only tumor cells are challenged.

In this study, we sought to evaluate the role of lactadherin, both in patient samples and present in sEVs secreted by tumor cells, as a possible molecular target that provides information on the prognosis of patients, which could suggest a possible combined treatment, including the blockade of lactadherin present in sEVs as an anti-metastatic option. In this way, future and needed steps are to evaluate directly in BC patients how the levels of this protein vary in plasmatic EVs, and to evaluate its relationship with patients’ prognosis and whether it could serve as a new therapeutic target, as something that brings us closer to the new concept of theragnostic.

## Conclusions

To our knowledge, these results strengthen the association between lactadherin levels and poor outcomes in patients with BC. Furthermore, our study provides the first evidence of the role of lactadherin in metastatic BC cell-secreted sEVs: (i) as a promoter of metastatic capacity in less aggressive recipient cells in vitro, and ii) its effects on the formation of ascites and metastatic tumor nodules in vivo. These results increase our understanding of the role of lactadherin in sEVs as a promoter of metastatic capacity, which can be used as a therapeutic option for BC and other malignancies.

### Supplementary Information

**Additional file 1: Figure S1.** Lactadherin (MFGE8) mRNA expression in different BC cell lines analyzed in this study. (Relative to Fig. 2). (**A-B**) TCGA data from two datasets were downloaded from UCSC Xena database and *MFGE8* expression levels were evaluated between different BC cell lines (**A**) Neve 2006 dataset; (**B**) Heiser 2012 dataset. (**C**) *MFGE8* expression in several BC cell lines (including those analyzed in this study) was also analyzed using DepMap expression analysis tool. BC cell lines were classified as ERpos/HER2pos, ERpos/HER2neg, ERneg/HER2pos and ERneg/HER2neg. Lactadherin expression in MCF10A normal mammary epithelial cells was included as control. **Figure S2.** Gating strategy for FACS analysis. (Relative to Fig. 3). SSC (log) vs FSC (log) to gate viable tumor cells; SSC (area) vs FSC (area) to gate single cells only; SSC (log) vs FITC (log) to gate and analyze lactadherin expression (stained with AF-488 conjugated antibody). **Figure S3.** Size distribution analysis of sEVs isolated by different BC cell lines. (Relative to Fig. 4). (A) Concentration of particles per mL of each sEVs analyzed, as determined by NTA analysis. (B) sEVs mean, and (C) mode size of sEV secreted by BC cell lines. (D) Size distribution of each sEVs analyzed. Each graph shows values ± SD. MCF10A normal mammary epithelial cells were included as control. **Figure S4.** Tumoroids retain their viability after passing through a 70 µm filter. (Relative to Fig. 6). The spheres/tumoroids retained on the filter were recovered and plated onto a 12-well adhesion plate. After 24 h, the adhered spheres were fixed with 4% PFA in 1X PBS for 10 min, washed and stained with DAPI 1:300 for 10 min. Finally, spheres were washed 3 times with 1X PBS and visualized and recorded under the same microscope to confirm that the recovered spheres remain viable. Representative images of MDA-MB-231 tumoroids are shown. **Figure S5.** sEV-MDA231 promotes mesenteric tumor growth, but does not increase total mass of other peritoneal and non-peritoneal organs. (Relative to Fig. 8). (A) The peritoneal treatment with sEV-MDA231 promotes tumor growth in MDA-MB-231 peritoneally-inoculated mice mesentery. Previous lactadherin blockade in those sEVs partially abrogates their effect, but that was not statistically significant. (B-D) Total mass of other organs such as liver (**B**), kidneys (**C**) and lungs (**D**) was not affected by the treatments. **Figure S6.** Lactadherin Z-score (enrichment) on BCC (Breast Cancer Cells) and EVs proteomic data (116). (Relative to discussion). Z-score was calculated from proteomic data available on Rontogianni et al., 2019 [96]. Mean z-score of grouped TNBC, HER2 + and HER2- (ER + /PR +) cells (red bars) and EVs (blue bars) were plotted.

## Data Availability

Raw and public data analyses are available under request.

## Abbreviations

BC: Breast cancer
EVs: Extracellular vesicles
NTA: Nano tracking analysis
sEVs: Small extracellular vesicles
TCGA: The Cancer Genome Atlas
TEM: Transmission electron microscopy
TNBC: Triple-negative breast cancer
WB: Western blot
