# Supplementary material for: Lactadherin immunoblockade in small extracellular vesicles inhibits sEV-mediated increase of pro-metastatic capacities
Source: Biol Res. 2024 Jan 3;57:1. doi: 10.1186/s40659-023-00477-8 (PMC10763369; doi:10.1186/s40659-023-00477-8)
Supplement: Supplementary file 1 — Additional file 1: Figure S1. Lactadherin (MFGE8) mRNA expression in different BC cell lines analyzed in this study. (Relative to Fig. 2). (A-B) TCGA data from two datasets were downloaded from UCSC Xena database and MFGE8 expression levels were evaluated between different BC cell lines (A) Neve 2006 dataset; (B) Heiser 2012 dataset. (C) MFGE8 expression in several BC cell lines (including those analyzed in this study) was also analyzed using DepMap expression analysis tool. BC cell lines were classified as ERpos/HER2pos, ERpos/HER2neg, ERneg/HER2pos and ERneg/HER2neg. Lactadherin expression in MCF10A normal mammary epithelial cells was included as control. Figure S2. Gating strategy for FACS analysis. (Relative to Fig. 3). SSC (log) vs FSC (log) to gate viable tumor cells; SSC (area) vs FSC (area) to gate single cells only; SSC (log) vs FITC (log) to gate and analyze lactadherin expression (stained with AF-488 conjugated antibody). Figure S3. Size distribution analysis of sEVs isolated by different BC cell lines. (Relative to Fig. 4). (A) Concentration of particles per mL of each sEVs analyzed, as determined by NTA analysis. (B) sEVs mean, and (C) mode size of sEV secreted by BC cell lines. (D) Size distribution of each sEVs analyzed. Each graph shows values ± SD. MCF10A normal mammary epithelial cells were included as control. Figure S4. Tumoroids retain their viability after passing through a 70 µm filter. (Relative to Fig. 6). The spheres/tumoroids retained on the filter were recovered and plated onto a 12-well adhesion plate. After 24 h, the adhered spheres were fixed with 4% PFA in 1X PBS for 10 min, washed and stained with DAPI 1:300 for 10 min. Finally, spheres were washed 3 times with 1X PBS and visualized and recorded under the same microscope to confirm that the recovered spheres remain viable. Representative images of MDA-MB-231 tumoroids are shown. Figure S5. sEV-MDA231 promotes mesenteric tumor growth, but does not increase total mass of other peritone [file 40659_2023_477_MOESM1_ESM.docx]

**Supplementary Figures**

**
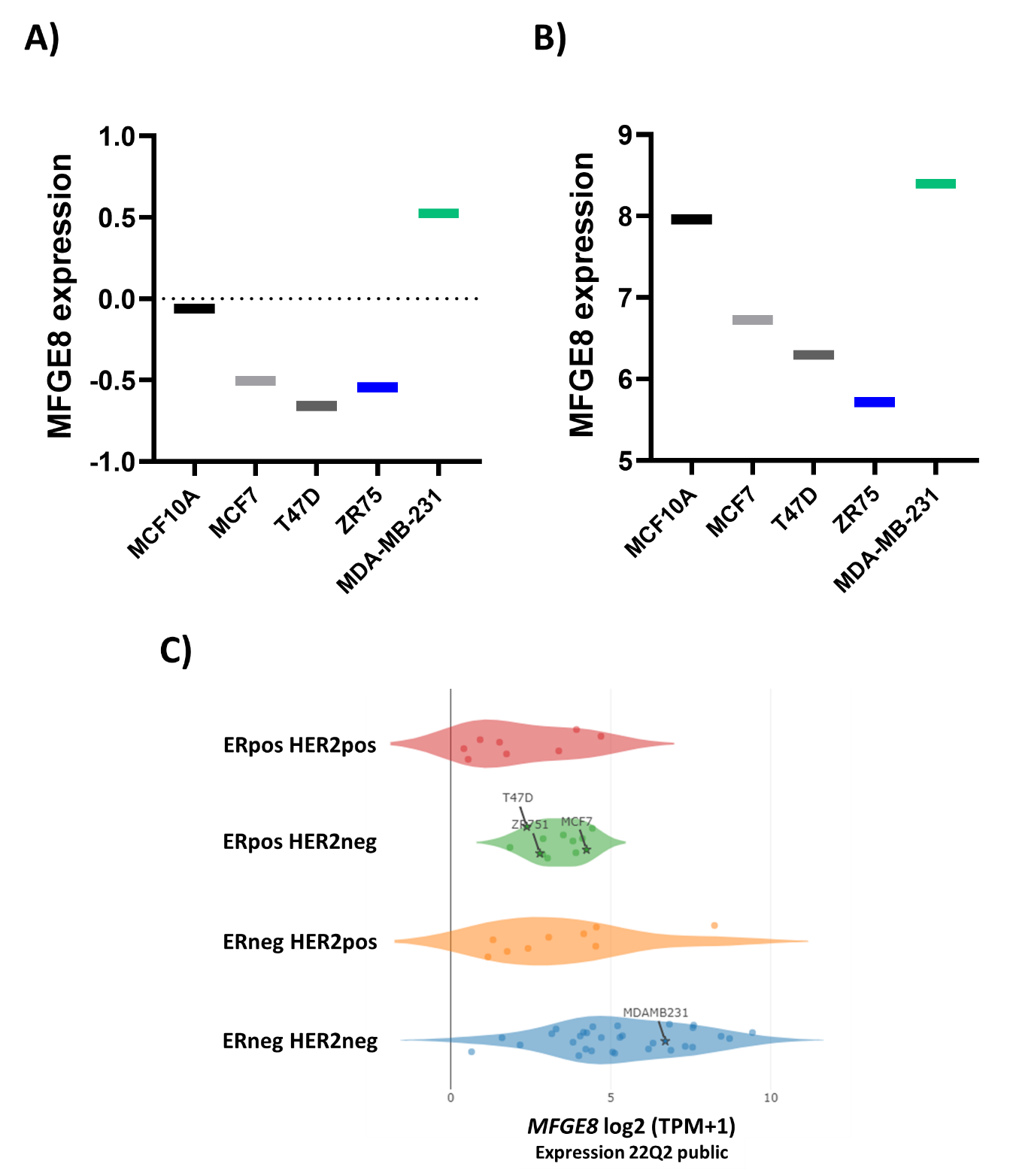
**

**Supplementary Figure 1. Lactadherin (MFGE8) mRNA expression in different BC cell lines analyzed in this study.** (Relative to Figure 2). (A-B) TCGA data from two datasets were downloaded from UCSC Xena database and *MFGE8* expression levels were evaluated between different BC cell lines (A) Neve 2006 dataset; (B) Heiser 2012 dataset. (C) *MFGE8* expression in several BC cell lines (including those analyzed in this study) was also analyzed using DepMap expression analysis tool. BC cell lines were classified as ERpos/HER2pos, ERpos/HER2neg, ERneg/HER2pos and ERneg/HER2neg. Lactadherin expression in MCF10A normal mammary epithelial cells was included as control.


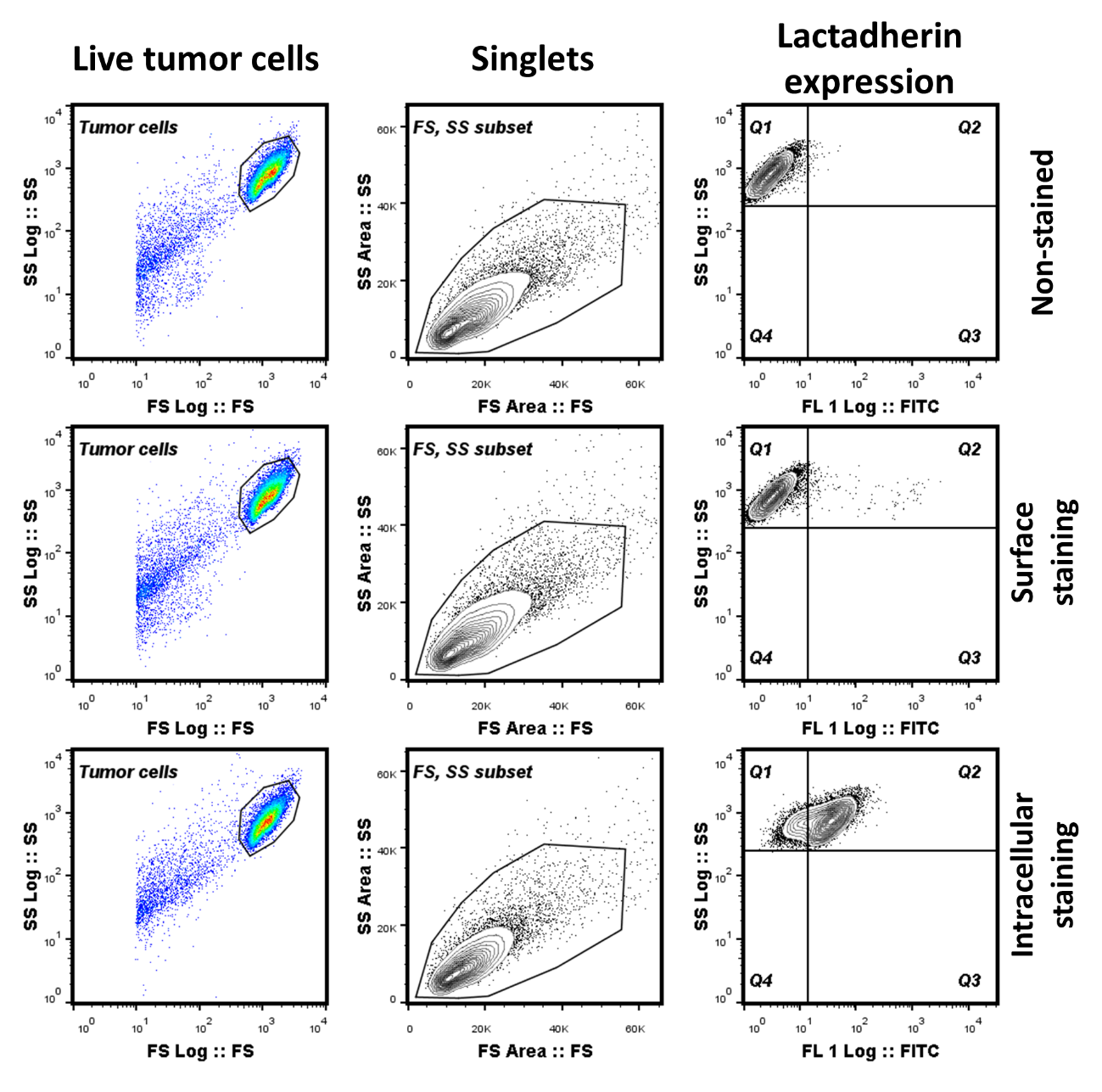


**Supplementary Figure 2. Gating strategy for FACS analysis.** (Relative to Figure 3). SSC (log) vs FSC (log) to gate viable tumor cells; SSC (area) vs FSC (area) to gate single cells only; SSC (log) vs FITC (log) to gate and analyze lactadherin expression (stained with AF-488 conjugated antibody).


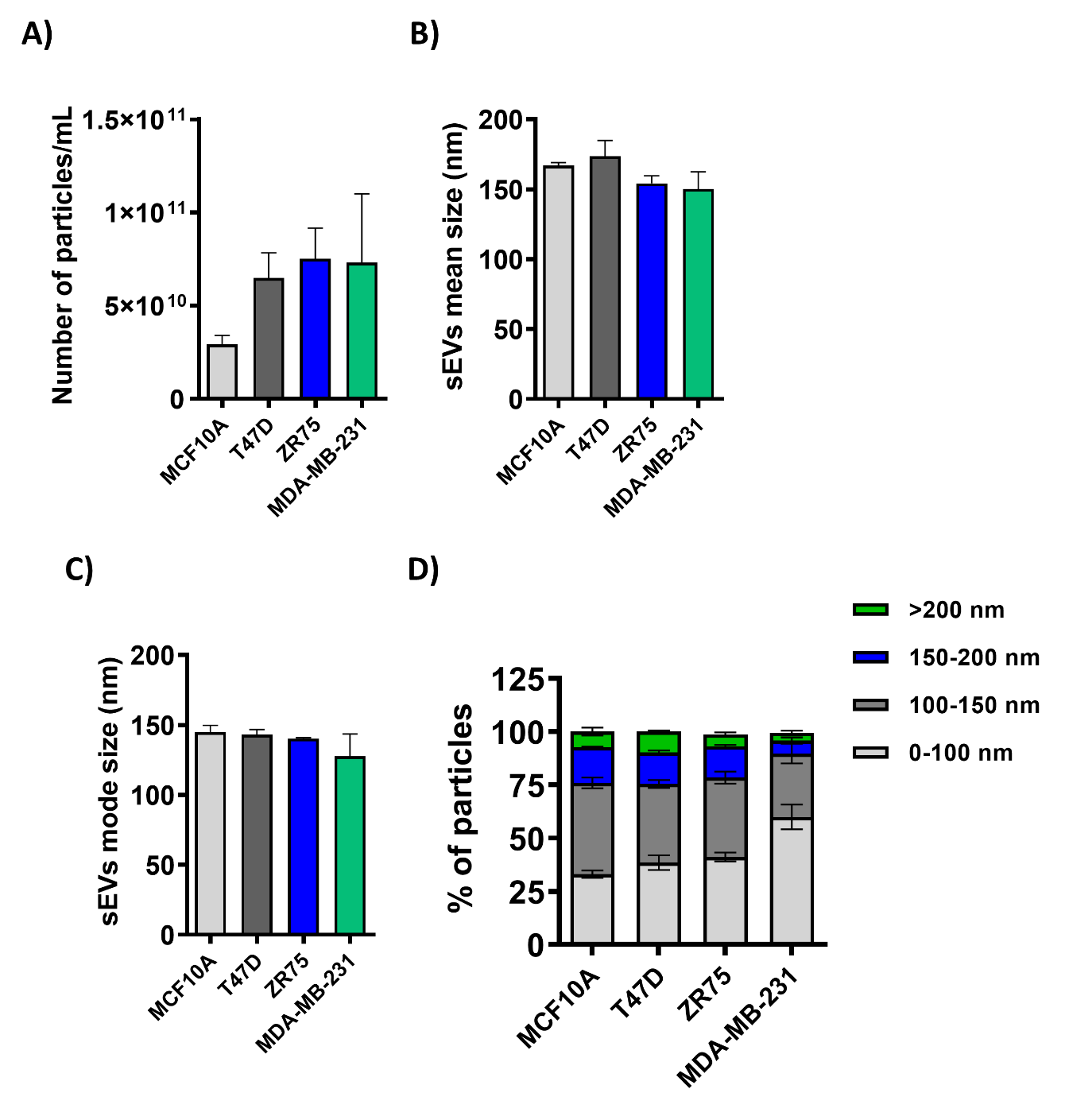


**Supplementary Figure 3. Size distribution analysis of sEVs isolated by different BC cell lines.** (Relative to Figure 4). (A) Concentration of particles per mL of each sEVs analyzed, as determined by NTA analysis. (B) sEVs mean, and (C) mode size of sEV secreted by BC cell lines. (D) Size distribution of each sEVs analyzed. Each graph shows values ± SD. MCF10A normal mammary epithelial cells were included as control.


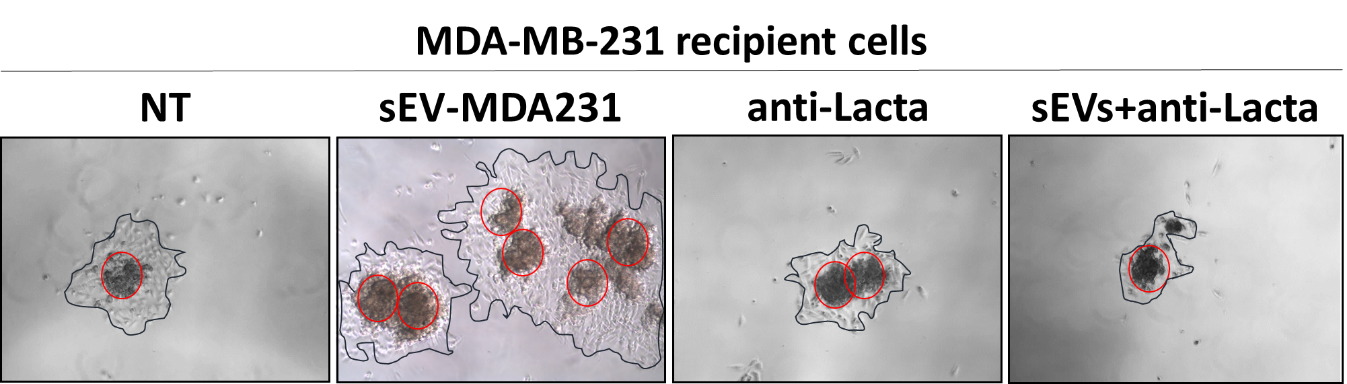


**Supplementary Figure 4. Tumoroids retain their viability after passing through a 70 µm filter.** (Relative to Figure 6). The spheres/tumoroids retained on the filter were recovered and plated onto a 12-well adhesion plate. After 24 h, the adhered spheres were fixed with 4% PFA in 1X PBS for 10 min, washed and stained with DAPI 1:300 for 10 min. Finally, spheres were washed 3 times with 1X PBS and visualized and recorded under the same microscope to confirm that the recovered spheres remain viable. Representative images of MDA-MB-231 tumoroids are shown.


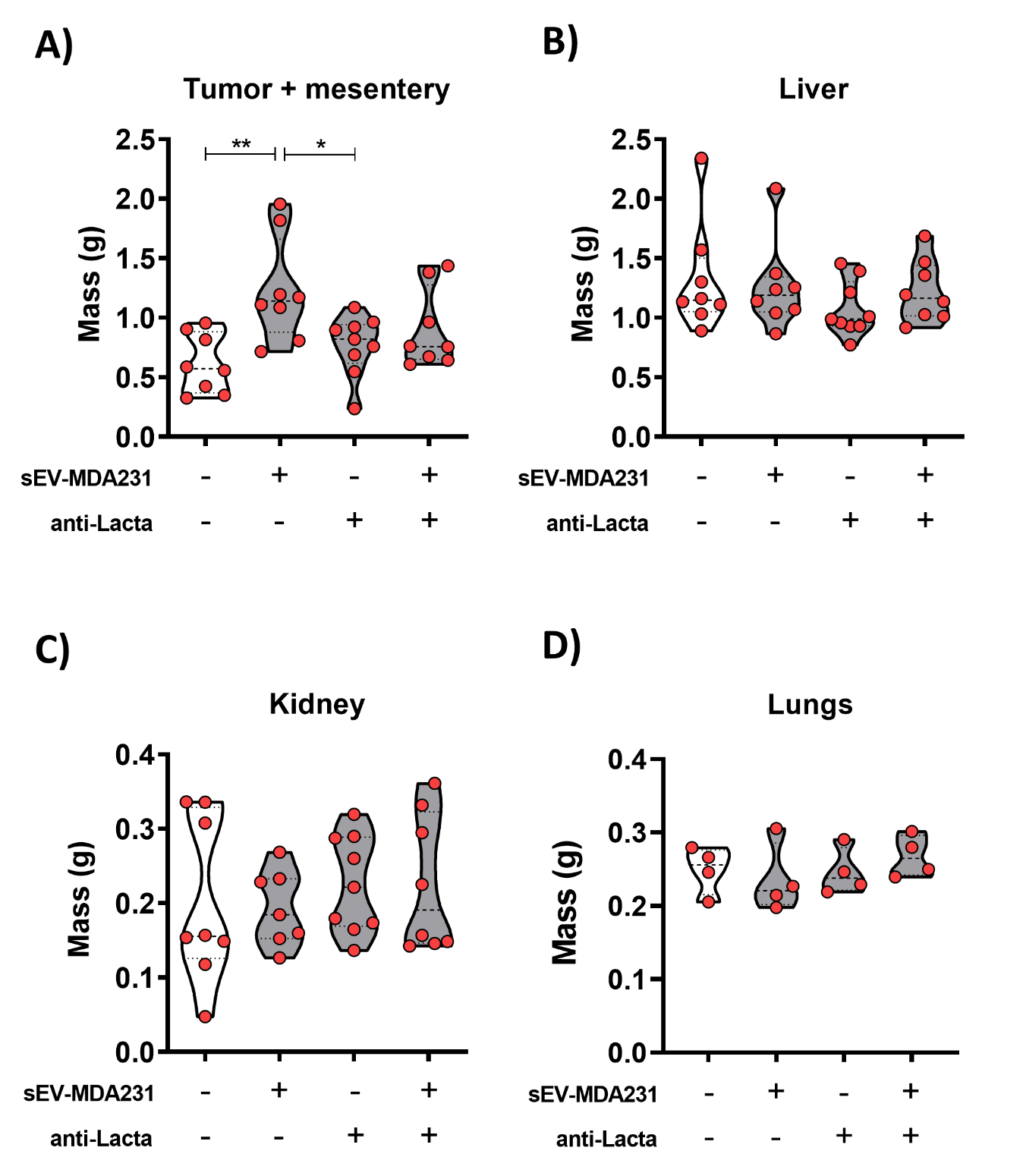


**Supplementary Figure 5. sEV-MDA231 promotes mesenteric tumor growth, but does not increase total mass of other peritoneal and non-peritoneal organs.** (Relative to Figure 8). (A) The peritoneal treatment with sEV-MDA231 promotes tumor growth in MDA-MB-231 peritoneally-inoculated mice mesentery. Previous lactadherin blockade in those sEVs partially abrogates their effect, but that was not statistically significant. (B-D) Total mass of other organs such as liver (B), kidneys (C) and lungs (D) was not affected by the treatments.

**
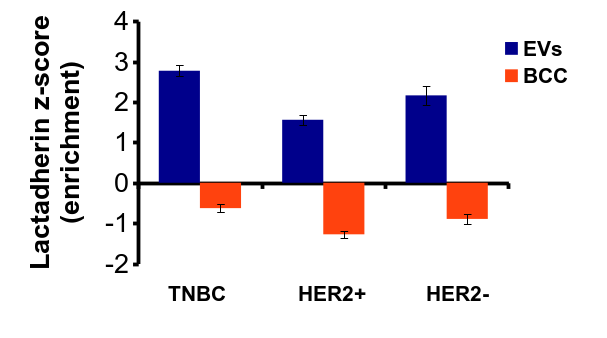
Supplementary Figure 6. Lactadherin Z-score (enrichment) on BCC (Breast Cancer Cells) and EVs proteomic data (116).** (Relative to discussion). Z-score was calculated from proteomic data available on Rontogianni et al, 2019 [96]. Mean z-score of grouped TNBC, HER2+ and HER2- (ER+/PR+) cells (red bars) and EVs (blue bars) were plotted.
